# Cross-cultural adaptation and validation of the Igbo language version of the stroke-specific quality of life scale 2.0

**DOI:** 10.11604/pamj.2020.37.111.19557

**Published:** 2020-10-01

**Authors:** Marufat Oluyemisi Odetunde, Adesola Christiana Odole, Nse Ayooluwa Odunaiya, Nurain Akinjide Odetunde, Emmanuel Chiebuka Okoye, Chidozie Emmanuel Mbada, Joseph Onuwa Umunnah, Aderonke Omobonike Akinpelu

**Affiliations:** 1 Department of Medical Rehabilitation, Obafemi Awolowo University, Ile-Ife, Nigeria,; 2 Department of Physiotherapy, College of Medicine, University of Ibadan, Ibadan, Nigeria,; 3 General Outpatient Department, Wesley Guild Hospital, Ilesha, Osun State, Nigeria,; 4 Department of Medical Rehabilitation, Faculty of Health Sciences and Technology, Nnamdi Azikiwe University, Nnewi Campus, Anambra State, Nigeria

**Keywords:** Cross-cultural adaptation, Igbo language, SS-QoL, validity, reliability

## Abstract

**Introduction:**

availability of the Stroke-Specific Quality of Life scale 2.0 (SS-QoL(E)) in Yoruba and Hausa, two of the three major indigenous languages in Nigeria have the potential to promote its uptake among these population, however, its non-availability in the Igbo languages makes its use restrictive among the south-eastern Nigerians. This study was aimed at cross-culturally adapting and assessing validity and reliability of the Igbo version of the SS-QoL.

**Methods:**

the SS-QoL(E) was cross-culturally adapted to Igbo following the American Association of Orthopaedic Surgeons’ guideline. This involved forward and back-translations, expert committee review, pretesting and cognitive debriefing interview to produce the final Igbo version, SS-QoL(I). The validity and reliability test involved 50 consenting Igbo stroke survivors. The construct validity was assessed by administering SS-QoL(E) and SS-QoL(I) on all 50 respondents, while SS-QoL(I) was re-administered at 7-day interval to assess test-retest reliability. Each scale was administered in random order. Data were analysed using Spearman’s correlation, Wilcoxon’s signed-rank test, Cronbach’s alpha, Intra-class Correlation Coefficient (ICC), independent t-test and one-way ANOVA at p<0.05.

**Results:**

respondents’ domains scores on SS-QoL(E) and SS-QoL(I) did not differ significantly except in mobility and work (r=0.58 to 0.87; p=0.001). Cronbach’s alpha was 0.69 to 0.87 for domains scores. The ICC ranged from 0.48 to 0.84, while no significant differences was found across different age groups or gender for the domains or overall scores of SS-QoL(I).

**Conclusion:**

the Igbo version of the SS-QoL has limited alterations from the original version and has moderate to excellent validity and reliability values.

## Introduction

Surviving a stroke, the second leading cause of death and a major cause of disability worldwide, can be a long-term process that affects many aspects of a person’s life [1,2]. Stroke is an important cause of morbidity and mortality with rising incidence in black Africans and other low- and middle-income countries where cardiovascular risk factors are on the increase [3]. In the West African sub-region, stroke is the leading cause of adult neurological admissions and accounted for 65% of such admissions [4,5]. In Nigeria, stroke is responsible for 0.9%-4% of total hospital admissions and 5%-45% of neurological admissions [6]. Stroke survivors (SSV) usually lose functionality and in Nigerian Africans, stroke impairs all facets of health-related quality of life (HRQoL) particularly in domains such as social, emotional, cognitive, physical and economic rendering them as burdens to their families and communities [7]. The more the severity of a stroke, the higher the disability and the lower the QoL of the SSV [8,9]. Consequently, stroke has detrimental effects on both short and long-term HRQoL of the SSV while disability resulting from stroke is a strong determinant of HRQoL [10,11].

Stroke survivors have impaired or decreased quality of life (QoL) on long term basis even among those who have no post-stroke disability [12,13]. With continued rehabilitation, however, improvements in functional status are possible and contribute to improve the QoL of SSV [12]. Treatment for stroke should therefore improve quality of life (QoL) by reducing the long-term consequences of the event. In chronic conditions such as stroke, evaluation of health-related quality of life (HRQoL) as a tool to assess changes in patient health from the patients’ perspective is vital to assess the domains which are directly impacted by the disease [14]. Patient-reported outcome measures have therefore been used to supplement clinical decisions made from clinician-based outcome measures [15]. There are conventional clinical scales that measure the treatment efficacy but these do not always capture dimensions of health that impact the QoL of SSV. Various specific instruments for assessing post-stroke HRQoL offer the advantage of assessing domains relevant to stroke, such as vision, language and use of paretic upper extremity. These, however, are not available in all languages. The Stroke-Specific Quality of Life scale version 2.0 (SS-QoL 2.0) is a comprehensive stroke outcome measure designed to capture multiple impacts of stroke in SSV. It comprises 49 items in 12 domains of self-care, vision, language, mobility, work, upper extremity function, thinking, personality, mood, family roles, social roles and energy [16]. The SS-QoL 2.0 has been cross-culturally adapted to many languages. These include Spanish [17], Danish [18], Chinese [19], German [20], Brazilian [21], Turkish [22], Persian [23] and Arabic [24].

Cross-cultural adaptation involves both translation and cultural adaptation concerns in the process of preparing a questionnaire for use in a different setting from what it was developed for [25]. It is the deliberate modification of some features of a questionnaire to better fit a particular target population [26]. The process ensures the attainment of semantic, idiomatic, experiential and conceptual equivalence between the source and the target questionnaires and enables comparisons and exchange of information across different populations with varying cultures and languages [27]. Nigeria is a multilingual nation with Hausa, Igbo and Yoruba as the three major languages. Igbo is the principal native language of the Igbo people, an ethnic group of south-eastern Nigeria. It is spoken by about 27 million speakers mostly from eastern Nigeria and minority language in Equatorial Guinea [28,29]. Igbo is one of the most widely spoken languages of West Africa and belongs to the Benue-Congo group of the Niger-Congo language family [28]. There are approximately thirty Igbo dialects which vary considerably with the standard literary form that is based on the Owerri and Umuahia dialects [28]. The primary Igbo States in Nigeria are Anambra, Abia, Ebonyi, Enugu and Imo States. Igbo speakers are typically bilingual, speaking English as well. Prominent Igbo communities outside Africa include Finland, Malaysia, London, UK, Houston, Atlanta and Washington DC, USA [29].

The SS-QoL 2.0 has been cross-culturally adapted into Yoruba [30] and Hausa [31], two of the three major Nigerian languages and validated [31,32]. Incidentally, the Yoruba version was the first African language version of SS-QoL. Non-availability of Igbo version of SS-QoL 2.0 might have limited its utilization in Nigeria especially among Igbo-speaking stroke survivors who are not literate in English language. The availability of translated and validated measures in the local languages is necessary to promote the use in a specific country. Hence, SS-QoL 2.0 in indigenous Igbo language will ensure that Igbo SSV who are not literate in English are not excluded from assessment of the QoL domains which directly impact them. This study was aimed at cross-culturally adapting and assessing validity and reliability of the Igbo version of the SS-QoL.

## Methods

**Cross-cultural adaptation of SS-QoL 2.0 into Igbo language:** the English Version of SS-QoL 2.0 was taken through forward translation to Igbo language by two independent bilingual translators following the guidelines of American Association of Orthopaedic Surgeons (AAOS) [25]. The translators who are proficient in English and Igbo languages and whose mother tongue is Igbo produced first and second translations (T1 and T2). One of the translators is a lecturer of physiotherapy; the second one is a teacher of Igbo language in a secondary school with more than ten years’ experience. The secondary school teacher was provided with information on the purpose and the concept involved in the SS-QoL 2.0 scale; however, the other translator was not given any information. The two translators met to review their translations (T1 and T2) and a consensus translation (T1, 2) was produced. A third bilingual translator (a lecturer of physiotherapy in a Nigerian University (Nnamdi Azikiwe University, Awka, Anambra State) domiciled among the Igbo speaking population) whose mother tongue is Igbo served as the scribe during the consensus translation. The consensus Igbo translation (T1, 2) was back translated to English by two independent translators who were totally blinded to the process of forward translation. The first back-translator is an Igbo language lecturer at the Nigeria University. The second back-translator is a translator at a radio station in Awka. Two back translations BT1 and BT2 were produced from this process. In line with the guideline, an expert committee was set up for the purpose of this study. The committee comprised six members: one forward translator (the secondary school teacher) and one back translator (the physiotherapy clinician), a lecturer of physiotherapy and two physiotherapy clinicians with many years of experience treating stroke survivors and the lead author who served as the scribe. Both the lecturer and clinician have previous experience in outcomes measurement research. The committee considered the instructions, response options and the items one after the other on the English version of SS-QoL, T1, T2, T12, BT1 and BT2 to assess consistency between the English version and Igbo translations of the SS-QoL 2.0 scale. At the end of the expert committee meeting, the pre-final Igbo version of SS-QoL 2.0 (PFI) was produced.

**Findings from expert committee meetings:** the expert committee observed that almost all the items in Igbo translations of SS-QoL 2.0 scale captured the concept of interest as provided in the English version. However, five expressions/items were identified to need further clarification and adjustment (Annex 1). These were addressed at the expert committee meeting. At the end of the meeting, PFI was produced.

**Findings from pre-testing and cognitive debriefing interview:** the PFI was pretested on fourteen stroke survivors (7 males and 7 females) who were indigenous Igbo speakers. This was followed by cognitive debriefing interview by the research assistant in which respondents were asked if all relevant areas were covered by the scale and if all the items were relevant to them. They were also asked about the clarity or ambiguity of the items and response options. All respondents understood the items and response options on the PFI and opined that all the items were relevant and no important area was left out. Four males out of all the 14 respondents indicated that item SC1 ‘did you have trouble preparing food’ was not applicable to them. This is because food preparation is considered as the duty of females and males are not normally involved in the process. Female respondents indicated there were not enough examples on female dressing on item SC4 and the example ‘tie your wrapper or scarf/head gear’ was added to the item in another expert committee meeting. Their comments and answers to the interview were considered at the second expert committee meeting where the PFI was adjusted accordingly to produce the final Igbo version of the Stroke-Specific Quality of Life scale 2.0 (SS-QoL(I)). A physiotherapist whose mother tongue is Igbo was trained as research assistant to facilitate the process of cross-cultural adaptation of the SS-QoL 2.0 into Igbo language.

**Psychometric testing of Igbo version of the SS-Qol 2.0:** for the validation of SS-QoL(I), demographic and clinical data of respondents were obtained using a proforma. Five indigenous Igbo physiotherapists, one in each centre trained as research assistants administered the English and Igbo versions of SS-QoL 2.0 on the entire fifty respondents. The scales were administered in a random order to assess concurrent and known-group validity of SS-QoL(I). The SS-QoL(I) was re-administered on the respondents at a 7-day interval to assess its test-retest reliability. We hypothesised that domains of English and Igbo versions of SS-QoL 2.0 would correlate significantly; and each item would correlate strongly with its own domain.

**Respondents:** this study was conducted at the outpatient physiotherapy departments of selected secondary and tertiary health institutions in the south-eastern States of Nigeria. Fifty consenting Igbo stroke survivors who were literate in English language and were receiving physiotherapy participated in the study. Respondents included were indigenous Igbo stroke survivors, with a diagnosis of ischemic or haemorrhagic stroke of at least one month [16], with first attack or recurrent stroke having varying levels of motor impairment, stroke duration and cognitive abilities that understood the items on the questionnaires and chose a response option for each item. This is in line with recommendations of Lin *et al*. [33] in order to increase the external validity and generalizability of the results of the study. Exclusion criteria were stroke survivors with dysphasia that interfered with meaningful communication; those with co-morbidities that concurrently affect HRQoL and those on hospital admission. The study was approved by Health Research Ethics committee of University of Ibadan/University College Hospital Ibadan.

**Data analysis:** descriptive statistics of mean and standard deviation were used to analyse domains and overall scores on SS-QoL(I). Discriminant and concurrent validity of the SS-QoL(I) were tested by using the Spearman’s correlation method. Known-group validity by comparing domain scores with gender and age groups was tested using independent t-test and one-way ANOVA respectively. Intra-class correlation (ICC) was used to determine the test-retest reliability and Cronbach’s alpha values were determined for internal consistency of the SS-QoL(I). SPSS version 16.0 (Chicago IL SPSS Inc.) was used to analyse data. Level of significance was set at p<0.05.

## Results

Respondents’ mean age was 55.64±10.3 years with median stroke duration of 12 months while mean age at stroke onset was 54.85±11.50. Majority (78%) of the respondents in this study were aged 50 years and above at the onset of stroke. Sixty per cent had right hemiplegia/hemiparesis while 40% had left hemiplegia/hemiparesis. Fifty per cent of the respondents have had stroke for 6 months or less, 50% are of more than 6-month duration. Respondents’ mean overall score on the SS-QoL(E) (182.60±36.48) did not differ significantly from that of SS-QoL(I) (173.53±33.05). The scores on all the domains of both versions did not differ significantly, except in mobility and work domains. Respondents’ scores were highest on the language domain (20.6±5.7) for SS-QoL(E) and 20.8+5.92 for SS-QoL(I) and lowest in the work domain (7.5±3.23 for SS-QoL(E) and 8.2±3.5 for SS-QoL(I). There were moderate to high (r=0.58 to 0.87) and significant correlations between overall and domains’ scores on the SS-QoL(E) and SS-QoL(I). The ICC on SS-QoL(I) was 0.91 for the overall score while domains’ score ranged from 0.50 to 0.84. Cronbach’s alpha values for eleven out of the twelve domains of SS-QoL(I) ranged from 0.69 to 0.87. Vision domain has Cronbach’s alpha value of 0.21. None of the domains or the overall score of SS-QoL(I) demonstrated significant floor effect (<20%) while 3 domains (vision, language and personality) showed significant ceiling effects (>20%). For the known-group validity of the SS-QoL(I) by gender and age, Table 1 shows the result of independent t-test comparison of domains and overall scores by gender. The result showed no significant gender difference in the domains and overall scores (p>0.05). Table 2 shows the result of one-way ANOVA comparison of domains and overall scores by age group. There were no significant differences in the mean domains and overall scores on SS-QoL(I) across various age groups (p>0.05). The details of item-domain correlations (discriminant validity) for SS-QoL(I) are presented in Table 3. The items showed a high level of item-domain correlations that ranged from 0.820 on vision domain to 0.979 on thinking domain. Correlations are interpreted as negligible (0.00 to 0.30), low (0.30 to 0.50), moderate (0.50 to 0.70), high (0.70 to 0.90) and very high (0.90 to 1.00) [34].

**Table 1:** independent t-test comparison of domains and overall score of the final Igbo version of the stroke-specific quality of life scale by gender

Domain	Gender			
	Male	Female		
	X̄±SD (n=26)	X̄±SD (n=24)	t-cal	p-value
SC	16.19 ± 5.15	17.71 ± 5.30	-1.03	0.93
V	13.77±2.16	13.38± 2.30	0.63	0.69
L	20.42± 6.18	21.29 ± 5.71	-0.51	0.45
M	20.15 ± 7.20	19.63 ± 7.35	0.26	0.74
W	6.92 ± 3.10	8.17± 3.34	-1.37	0.36
UE	14.77 ± 5.98	16.88± 5.56	-1.27	0.79
T	11.58 ±3.87	11.00 ± 3.83	0.53	0.73
P	9.50 ± 4.47	9.25 ± 4.40	0.20	0.99
MD	17.04 ± 5.60	16.50 ± 5.35	0.35	0.36
FR	9.00 ± 4.20	8.83 ± 4.06	0.14	0.95
SR	11.69 ± 5.97	9.83 ± 5.07	1.18	0.22
E	9.46 ± 4.14	8.58 ± 4.04	0.76	0.66
O	161.15 ± 41.01	161.04 ± 38.74	0.01	0.56

Alpha level was set at p <0.05. SC-self-care; V-vision; L-language; M-mobility; W-work; UE-upper extremity; T-thinking; P-personality; MD-mood; FR-family role; SR-social role; E-energy; O-overall

**Table 2:** one-way ANOVA comparison of the Igbo version of the stroke-specific quality of life scale 2.0 domains by age group

	Age groups					
SS-QoL domain	<50	50-59	60-69	>70		
	X̄ ± SD (n=12)	X̄ ± SD (n=25)	X̄ ± SD (n=10)	X̄ ± SD (n=5)	F-ratio	P-value
SC	19.42 ± 5.32	17.26 ± 5.25	16.00 ± 3.50	11.20 ± 3.90	3.52	0.02^*^
V	11.81 ± 2.23	13.87 ± 1.98	13.60 ± 2.01	11.60 ± 4.22	1.57	0.21
L	20.33 ± 7.13	21.91 ± 5.19	20.50 ± 5.81	17.80 ± 6.80	0.72	0.55
M	21.33 ± 7.25	18.49 ± 7.83	22.40 ± 4.58	18.40 ± 7.47	0.95	0.42
W	9.33 ± 3.47	7.17 ± 3.14	6.90 ± 2.81	6.00 ± 3.08	1.93	0.14
UE	18.00 ± 5.81	14.91 ± 5.70	15.80 ± 5.57	7.23 ± 3.23	0.84	0.48
T	11.42 ± 4.32	12.17 ± 3.56	10.20 ± 4.32	9.20 ± 1.79	1.20	0.32
P	8.17 ± 5.08	9.96 ± 4.49	10.00 ± 3.20	7.63 ± 8.40	0.57	0.64
MD	14.75 ± 6.00	18.17 ± 5.33	17.20 ± 5.29	14.40 ± 3.44	1.44	0.24
FR	7.50 ± 4.21	9.70 ± 4.18	9.30 ± 4.42	8.00 ± 2.35	0.86	0.47
SR	10.25 ± 6.41	11.78 ± 6.40	9.80 ± 3.55	9.60 ± 2.07	0.44	0.72
E	8.58 ± 4.40	8.78 ± 4.20	10.30 ± 3.65	8.80 ± 4.27	039	0.76
O	162.92 ± 44.04	164.91 ± 43.27	162.00 ± 31.38	137.80 ± 21.65	0.65	0.59

* Significant differences. Alpha level was set at p <0.05, SC-self-care; V-vision; L-language; M-mobility; W-work; UE-upper extremity; T-thinking; P-personality; MD-mood; FR-family role; SR-social role; E-energy; O-overall

**Table 3:** Spearman’s item-domain correlations (discriminant validity) of the Igbo version of the stroke-specific quality of life scale 2.0 (n=50)

SS-QoL domain	SC	V	L	M	W	UE	T	P	MD	FR	SR	E
SC1	0.902	0.209	0.390	0.471	0.642	0.560	0.172	0.107	0.340	0.360	0.362	0.362
SC2	0.876	0.307	0.344	0.456	0.613	0.544	0.121	0.118	0.302	0.312	0.332	0.332
SC4	0.893	0.210	0.444	0.460	0.650	0.664	0.186	0.062	0.243	0.286	0.249	0.249
SC5	0.911	0.143	0.508	0.442	0.651	0.595	0.139	0.038	0.315	0.364	0.321	0.321
SC8	0.829	0.107	0.376	0.389	0.596	0.490	0.168	0.075	0.308	0.278	0.271	0.271
V1	0.265	0.820	0.372	0.167	0.168	0.367	0.274	0.143	0.373	0.362	0.384	0.384
V2	0.145	0.874	0.252	0.089	0.218	0.298	0.172	0.177	0.296	0.329	0.327	0.327
V3	0.143	0.867	0.198	0.036	0.128	0.341	0.196	0.167	0.309	0.334	0.112	0.368
L2	0.531	0.381	0.941	0.259	0.448	0.242	0.265	0.220	0.266	0.300	0.128	0.267
L3	0.455	0.294	0.930	0.175	0.390	0.255	0.218	0.142	0.223	0.260	0.108	0.250
L5	0.455	0.250	0.937	0.243	0.341	0.301	0.212	0.265	0.273	0.336	0.113	0.285
L6	0.509	0.409	0.891	0.264	0.371	0.221	0.255	0.265	0.315	0.348	0.137	0.322
L7	0.547	0.273	0.894	0.235	0.454	0.313	0.247	0.213	0.234	0.254	0.154	0.275
M1	0.469	0.168	0.225	0.929	0.607	0.584	0.263	0.326	0.497	0.438	0.398	0.523
M4	0.453	0.084	0.098	0.899	0.530	0.578	0.237	0.288	0.467	0.435	0.397	0.509
M6	0.465	0.127	0.139	0.938	0.582	0.554	0.240	0.271	0.449	0.423	0.393	0.514
M7	0.419	0.194	0.202	0.869	0.565	0.582	0.247	0.283	0.464	0.428	0.405	0.558
M8	0.403	0.137	0.050	0.875	0.556	0.566	0.215	0.270	0.432	0.422	0.412	0.529
M9	0.395	0.085	0.185	0.808	0.509	0.534	0.225	0.229	0.436	0.375	0.373	0.507
W1	0.701	0.190	0.306	0.525	0.922	0.610	0.160	0.269	0.338	0.310	0.482	0.513
W2	0.653	0.138	0.437	0.584	0.941	0.592	0.225	0.313	0.379	0.316	0.483	0.512
W3	0.670	0.118	0.253	0.561	0.888	0.618	0.174	0.284	0.362	0.384	0.469	0.500
UE1	0.556	0.047	0.254	0.394	0.549	0.957	0.129	0.296	0.394	0.453	0.431	0.416
UE2	0.616	0.031	0.261	0.603	0.587	0.969	0.157	0.270	0.370	0.439	0.446	0.400
UE3	0.601	0.062	0.381	0.407	0.565	0.973	0.198	0.320	0.396	0.456	0.456	0.423
UE4	0.614	0.091	0.197	0.536	0.579	0.964	0.207	0.301	0.412	0.469	0.403	0.425
UE5	0.654	0.041	0.303	0.559	0.572	0.904	0.150	0.318	0.354	0.364	0.317	0.328
T2	0.213	0.224	0.318	0.148	0.187	0.256	0.964	0.485	0.636	0.486	0.581	0.567
T3	0.153	0.187	0.255	0.157	0.176	0.178	0.979	0.436	0.617	0.444	0.512	0.519
T4	0.322	0.258	0.296	0.292	0.301	0.180	0.963	0.440	0.581	0.445	0.453	0.512
P1	0.319	0.089	0.331	0.343	0.458	0.377	0.415	0.973	0.615	0.642	0.539	0.576
P2	0.320	0.145	0.394	0.310	0.376	0.306	0.450	0.972	0.589	0.616	0.521	0.567
P3	0.291	-0.008	0.320	0.301	0.385	0.247	0.436	0.969	0.625	0.618	0.513	0.586
MD2	0.427	0.103	0.418	0.361	0.251	0.430	0.603	0.606	0.960	0.738	0.589	0.715
MD3	0.339	0.078	0.413	0.356	0.266	0.424	0.642	0.598	0.970	0.771	0.609	0.739
MD6	0.403	0.279	0.420	0.354	0.280	0.412	0.646	0.573	0.953	0.779	0.627	0.752
MD7	0.442	0.103	0.448	0.488	0.364	0.409	0.637	0.612	0.955	0.770	0.564	0.710
MD8	0.399	0.159	0.325	0.358	0.305	0.375	0.625	0.595	0.947	0.730	0.509	0.657
FR5	0.473	0.022	0.319	0.434	0.419	0.463	0.466	0.581	0.720	0.961	0.620	0.603
FR7	0.456	-0.073	0.189	0.480	0.346	0.515	0.445	0.617	0.760	0.955	0.594	0.619
FR8	0.450	0.010	0.344	0.434	0.312	0.441	0.408	0.639	0.745	0.961	0.594	0.632
SR1	0.536	0.167	0.272	0.423	0.419	0.481	0.465	0.473	0.577	0.633	0.955	0.649
SR4	0.469	0.001	0.186	0.413	0.419	0.446	0.498	0.514	0.567	0.617	0.962	0.662
SR5	0.390	0.092	0.268	0.368	0.305	0.454	0.556	0.544	0.600	0.616	0.964	0.687
SR6	0.539	0.024	0.278	0.393	0.409	0.492	0.506	0.621	0.602	0.629	0.959	0.668
SR7	0.532	0.044	0.184	0.489	0.403	0.478	0.529	0.565	0.556	0.603	0.956	0.676
E1	0.275	-0.017	0.187	0.419	0.243	0.397	0.484	0.622	0.645	0.606	0.458	0.967
E2	0.326	0.079	0.266	0.540	0.341	0.436	0.534	0.568	0.732	0.649	0.537	0.974
E3	0.244	-0.004	0.170	0.373	0.248	0.495	0.567	0.563	0.755	0.666	0.326	0.958

r= 0.00 to 0.30 (negligible), 0.30 to 0.50 (low), 0.50 to 0.70 (moderate), 0.70 to 0.90 (high), 0.90 to 1.00 (very high). SC-self-care; V-vision; L-language; M-mobility; W-work; UE-upper extremity; T-thinking; P-personality; MD-mood; FR-family role; SR-social role; E-Energy

## Discussion

Igbo is one of the three major Nigerian languages spoken by 21% of the population [35]. It is spoken mainly in south-eastern States and in the north-east of Delta State and in the south-east of Rivers State by a total of about 18 million people of the Nigerian population [35] aside other parts of the world. The process of cross-cultural adaptation of SS-QoL 2.0 into Igbo language in this study revealed that one-step back-translation of consensus Igbo version of SS-QoL 2.0 was consistent with the English version for almost all the items. Cross-cultural adaptation process of the SS-QoL 2.0 to Igbo language, like that of Hausa and Yoruba languages, gave credence to AAOS guidelines for cross-cultural adaptation [25]. It shows that the various stages involved are important for a thorough cross-cultural adaptation process. The stage of back-translation served as validity check for the entire process while the stage of pretesting ensured complete equivalence between the target and source languages. Previous researches have also found back-translation worthwhile [18,36]. This study assessed construct, discriminant and known group validity, test-retest reliability and internal consistency of the SS-QoL(I). The SS-QoL(I) demonstrated moderate to excellent test-retest reliability and acceptable to good internal consistency on its domains. The SS-QoL(I) can discriminate between varying degrees of stroke severity, consistent with reports of Williams *et al*. [16] and Cruz-Cruz [37]. The known-group validity of the SS-QoL(I) indicated that there was no significant gender difference between the domains and overall scores. This is similar to report of Xie *et al*. [10] and Zalihic *et al*. [38]. All items in the SS-QoL(I) demonstrated strong correlations with its own hypothesized domain than with domains measuring other concepts.

The one-step back-translation conducted for SS-QoL(I) is different from the multi-step forward and back translations and expert committee meetings conducted during the cross-cultural adaptation of SS-QoL 2.0 scale into Yoruba reported by Akinpelu *et al*. [30]. The authors attributed the high number of forward-backward translation to dearth of certified English-Yoruba translators. The few differences in forward and back-translation of SS-QoL 2.0 into Igbo as seen in the use of words ‘defecating or urinating’ to describe the use of toilet in item SC8 and the use of expression ‘man and woman relationship’ for sex in item SR6 were based on cultural moral norms. Although the two English-Igbo translators in this study were not certified translators, they were experienced translators and this might be responsible for the consistent forward and back-translation obtained for SS-QoL 2.0 into Igbo language. The mean age of the stroke survivors who participated in the psychometric testing of SS-QoL(I) was 55.64±10.3 years and median stroke duration was 12 months. Over half of the SSV were aged 50 years and above at stroke onset. This supports the earlier reports that stroke occurs more frequently in middle age [1,21,30,33,39]. Although strokes can occur at any age, its risk increases with age [40] while stroke duration varies extensively among the survivors. This may be attributed to factors such as presence of co-morbidities, quality of care and access to care and rehabilitation, racial and ethnic disparities, perceived social support and psychological state among other factors [10]. Sex distribution of respondents in this study also indicated a male preponderance and this is in line with previous studies [21,30,33,41].

Reliability values of SS-QoL(I) are consistent with reports of Odetunde *et al*. [31,32] for Yoruba and Hausa versions of the SS-QoL respectively and in agreement with interrelatedness of the domains of SS-QoL 2.0 as observed by Hsueh *et al*. [42]. The values are also comparable with different versions of SS-QoL for various categories of stroke survivors [16,17,22,33,43]. The SS-QoL(I) measures the same construct as the English version as evidenced by the similarities in the overall and 10 out of 12 domains scores. The two domains (mobility and work) that demonstrated significant difference may be explained in terms of cultural differences between the two languages. This may imply that the Igbo version of the SS-QoL 2.0 is a valid translation of the English version. This is similar to reports of Akinpelu *et al*. [30] for Yoruba version and Odetunde *et al*. [31] for Hausa version of SS-QoL 2.0 respectively. The scale, however, could not identify improvements on vision, language, thinking and personality domains of stroke survivors beyond the level of the ceiling effect as the highest score fails to capture the most positive evaluation. These are similar to the reports of significant ceiling effects on all domains of SS-QoL 2.0 scale except thinking, social role and energy domains by Williams *et al*. [16]. The vision domain of SS-QoL(I) showed the highest ceiling effect, consistent with previous findings [16,20,42]. This may be because majority of the stroke survivors in this study who are above 50 years of age might be suffering from other visual problems due to advanced age as a result of which the SS-QoL(I) is not responsive to that domain.

Significant ceiling effect on the language domains of SS-QoL(I) may be because SS-QoL 2.0 does not assess severe language deficits among stroke survivors and since the respondents in this study did not have severe language deficits majority scored high on that domain. There is also the possibility that those with language and speech problems at the initial stage have recovered since majority of the respondents have had stroke for more than 6 months. The mood domain’s acceptable ceiling effect is also consistent with report of Hsueh *et al*. [42]. Female respondents had higher mean score in self-care, language, work and upper extremity domains while male respondents had higher mean score in mobility, mood and social role domains. In the Hausa and Yoruba versions of the SS-QoL male and female respondents had higher mean scores on various domains [31,32] which is consistent with the current study and opposes previous reports that females reported significantly lower post-stroke QoL [44,45]. We also found that the SS-QoL domains were associated with age. The mean domain scores were highest in the age group 50 and below followed closely by age group 50 to 59 on self-care, work and upper extremity domains. The age group 50 and below is made up of people in their productive years. Stroke recovery is expected to be faster in this age group than the older age group since advancing age is a poor prognostic factor for recovery from stroke [46,47]. Hence recovery of motor function demonstrated by high scores in the physical function domains is expected. Comparably high mean scores were recorded for age group 60 to 69 on personality and energy domains and age group 50 to 59 on mood domain.

The reported differences in the QoL scores among respondents in our study has demonstrated that age of stroke survivors was an important factor that determines their HRQoL [47,48]. Psychological function appeared to be less affected by age as demonstrated by SS-QoL scores on personality and mood domains, in line with reports of Alonso-Moran *et al*. [48] and Jeste *et al*. [49]. Items on SS-QoL(I) showed high to very high level of item-domain correlations. For example, item SC5 ‘did you have trouble taking a bath or a shower?’ had p-value of 0.911 with its own domain, while the same item had r-value of 0.143 with item V3 ‘did you have trouble seeing things off to side?’ on vision domain. This is similar to reports of Cruz-Cruz *et al*. [37] and Muus *et al*. [43] and describes the relevance of the items in their respective domains and the discriminant validity of the SS-QoL(I). This study has provided a cross-culturally adapted Igbo version of the SS-QoL 2.0 with good to excellent psychometric evidence for use in assessment of quality of life of Igbo stroke survivors who are not literate in English language. This will ensure that this group of stroke survivors is not excluded from assessment of health variables that are important and meaningful to them. The number of African language version of the SS-QoL 2.0 has thus been increased.

This study has some limitations that may need to be considered in interpreting and generalizing its findings. First, the generalizability of this study may be limited to stroke survivors in urban and semi urban communities. This is because stroke survivors from only departments of physiotherapy in health facilities within each of the capital city and other major cities of the south-eastern States of Nigeria were included. Second, respondents in this study received different rehabilitation programs throughout the duration of the study; further research is needed to assess the psychometric properties of the SS-QoL(I) for specific treatment programs on larger samples to provide further insights into the psychometric properties of the SS-QoL(I) on specific treatment program. There are possible differences in psychometric properties in patient-reported QoL outcomes due to the modes of administration [50], thus further research may be needed to study psychometric properties of the SS-QoL(I) using different modes of administration such as paper-and-pencil administration at home, via the mail and telephone interview. Lastly, in this study, the SS-QoL(I) was validated using the English version of SS-QoL. Other outcome measures with the advantage of assessing domains relevant to stroke should be used to compare the domains of SS-QoL(I) in order to further ascertain its validity with other established outcome measures.

## Conclusion

The Igbo version of the SS-QoL has limited alterations from the origin version with good to excellent validity and reliability values. The SS-QoL(I) is therefore recommended for assessing health-related quality of life among Igbo stroke survivors.

### What is known about this topic

- Cross-cultural adaptation involves translation and deliberate modification of some features of a questionnaire to better fit the culture of a particular target population;
- Cross-cultural adaptation enables comparisons of results across different studies both nationally and internationally;
- The stroke-specific quality of life scale has been cross-culturally adapted to many languages, with Yoruba as the first African language.

### What this study adds

- This study has provided a cross-culturally adapted Igbo version of the SS-QoL 2.0 with evidence of good to excellent validity and reliability and which can assess varying degrees of stroke severity among indigenous Igbo stroke survivors;
- This study has increased the number of African language versions of the SS-QoL available.

## Annex

**Annex 1**: findings from expert panel meetings on cross-cultural adaptation of SS-QoL 2.0 scale into Igbo language (PDF-299KB)
